# Alcohol-Induced Neuroadaptation Is Orchestrated by the Histone Acetyltransferase CBP

**DOI:** 10.3389/fnmol.2017.00103

**Published:** 2017-04-11

**Authors:** Alfredo Ghezzi, Xiaolei Li, Linda K. Lew, Thilini P. Wijesekera, Nigel S. Atkinson

**Affiliations:** ^1^Department of Biology, University of Puerto Rico, Río Piedras CampusSan Juan, Puerto Rico; ^2^Department of Neuroscience and Waggoner Center for Alcohol and Addiction Research, The University of Texas at AustinAustin, TX, USA

**Keywords:** synaptic homeostasis, addiction, *Drosophila*, chromatin remodeling, alcohol tolerance, CREB-binding protein

## Abstract

Homeostatic neural adaptations to alcohol underlie the production of alcohol tolerance and the associated symptoms of withdrawal. These adaptations have been shown to persist for relatively long periods of time and are believed to be of central importance in promoting the addictive state. In *Drosophila*, a single exposure to alcohol results in long-lasting alcohol tolerance and symptoms of withdrawal following alcohol clearance. These persistent adaptations involve mechanisms such as long-lasting changes in gene expression and perhaps epigenetic restructuring of chromosomal regions. Histone modifications have emerged as important modulators of gene expression and are thought to orchestrate and maintain the expression of multi-gene networks. Previously genes that contribute to tolerance were identified as those that show alcohol-induced changes in histone H4 acetylation following a single alcohol exposure. However, the molecular mediator of the acetylation process that orchestrates their expression remains unknown. Here we show that the *Drosophila* ortholog of mammalian CBP, *nejire*, is the histone acetyltransferase involved in regulatory changes producing tolerance—alcohol induces *nejire* expression, *nejire* mutations suppress tolerance, and transgenic *nejire* induction mimics tolerance in alcohol-naive animals. Moreover, we observed that a loss-of-function mutation in the alcohol tolerance gene *slo* epistatically suppresses the effects of CBP induction on alcohol resistance, linking *nejire* to a well-established alcohol tolerance gene network. We propose that CBP is a central regulator of the network of genes underlying an alcohol adaptation.

## Introduction

Alcohol addiction is a serious and debilitating condition characterized by compulsive and escalating alcohol use. Approximately 7% of adults per annum have an alcohol use disorder that requires treatment, and alcohol-related deaths are the third leading cause of preventable death in the U.S. (NIAAA, 2015). It is well established that alcoholism arises in part from alcohol-induced neuroadaptations that lead to a progressive increase in alcohol tolerance, to the emergence of physiological dependence and to associated withdrawal symptoms. Together, tolerance and physiological dependence are thought to contribute to the uncontrollable urge to consume alcohol through dysregulation of brain reward systems (Koob and Le Moal, 2001). These responses are produced by some of the earliest adaptations to alcohol. Although reversible, these adaptations are likely related to, or to contribute to, the adaptations produced by chronic exposure that generate the hallmarks of alcoholism—such as alcohol preoccupation, craving and a loss of control with respect to alcohol consumption.

Tolerance and withdrawal are highly conserved responses to alcohol that have been studied in many animal model systems. Because adult *Drosophila* do not acquire metabolic tolerance (Scholz et al., 2000), they are an ideal model system to study the neuronal adaptations that underlie functional alcohol tolerance. In *Drosophila*, alcohol tolerance and withdrawal responses closely recapitulate the mammalian response. A single sedative exposure to alcohol results in an increase in alcohol resistance that lasts over 10 days (Cowmeadow et al., 2005; Krishnan et al., 2016). In parallel with the development of tolerance, flies acquire mechanistically-related withdrawal symptoms (alcohol-induced neuronal hyperexcitability) that are similar to those described in humans after heavy alcohol use (Bayard et al., 2004; Ghezzi et al., 2014). Understanding the inceptive adaptations elicited by a single alcohol exposure is thus critical for understanding the underlying processes behind prolonged use.

In previous studies, we have linked the development of alcohol tolerance and alcohol withdrawal to an increase in expression of the *slo* gene. In both flies and humans the *slo* gene encodes BK-type Ca^2+^ activated K^+^ channels. Because these are the highest conductance K^+^ channel encoded in animals, a small change in activity can significantly alter signaling properties (reviewed in Latorre et al., 1989; Gribkoff et al., 2001). We found that up-regulation of BK channel gene expression by alcohol can by itself generate a substantial degree of tolerance. This adaptation represents a homeostatic process that directly opposes the sedative effect of the drug. After alcohol clearance however, this same adaptation leads to a withdrawal state characteristic of physiological drug dependence (Ghezzi et al., 2010, 2014).

Histone acetylation is a common method used by eukaryotes to enhance transcription initiation. It does so by: (1) controlling accessibility—acetylation directly promotes structural changes in chromatin that are favorable for transcription (decondensation); (2) by serving as a binding site for remodeling enzymes and transcription factors needed for transcription initiation; and (3) by occlusion—making a lysine residue unavailable for the addition of repressive histone modification (Choi and Howe, 2009; Galvani and Thiriet, 2015).

Previously, we characterized the time course of histone acetylation changes across the 7 kb *slo* promoter region to monitor how the *slo* gene responds to alcohol sedation (Wang et al., 2007). This helped us to identify DNA regulatory elements that control gene activity and led to the demonstration that the CREB transcription factor was required for alcohol-induced *slo* expression (Wang et al., 2009). We also surveyed the genome for other genes with a similar alcohol-induced histone H4 acetylation profile in order to identify additional genes that like *slo*, respond to alcohol (Ghezzi et al., 2013). In this endeavor, our focus was only on genes involved in producing tolerance and so we only examined genes whose acetylation status was similarly affected by two different sedative drugs—ethanol and benzyl alcohol. These drugs produce mutual cross-tolerance by overlapping or identical mechanisms. By restricting our focus we were able to identify a number of genes that had large effects on tolerance. One of the alcohol-responsive genes was *nejire*, the *Drosophila* gene that encodes CBP—a histone acetyltransferase. The working hypothesis that underlies the current article is that the alcohol-induced histone acetylation, which is tightly correlated with the induction of genes important for producing alcohol tolerance, is produced, at least in part, by *Drosophila* CBP; and that alcohol-induction of CBP is important for molecular and behavioral responses to alcohol.

## Materials and Methods

### Fly Stocks

*Drosophila* stocks were raised on standard cornmeal agar medium in a 12/12 h light/dark cycle. For all assays, newly enclosed flies were collected over a 2-day interval and studied 3–5 days after collection. All stocks used in this study are listed in Table 1.

**Table 1:** ***Drosophila* stocks used in this study**.

Genotype	Source
Canton S [CS]—wild type	BDSC (1)^a^
w* nej^3^/FM7c ; + ; +	BDSC (3729)^a^
w* ; + ; P{hs-nej+}1	BDSC (3730)^a^
*y*^1^, *nej*^Q7^, *v*^1^, *f*^1^/Dp(1;Y)FF1, *y*^+^/C(1)DX, *y*^1^, *w*^1^, *f*^1^ ; + ; +	BDSC (5292)^a^
*y*^1^, w* ; + ; Mi{MIC}*slo*^MI02233^/TM3, *Sb*^1^, *Ser*^1^	BDSC (37572)^a^
P{Hsp70-Gal4^DBD^:*Rpd3*}/FM6 ; + ; +	Gift from B.R. Calvi^b^
+ ; + ; *slo*^UAS-6b-L^	Generated in this study
+ ; + ; *slo*^6b-L^	Generated in this study
P{Hsp70-Gal4^DBD^:*Rpd3*}/FM6; + ; *slo*^UAS-6b-L^	Derived from crossing
P{Hsp70-Gal4^DBD^:*Rpd3*}/FM6 ; + ; *slo*^6b-L^	Derived from crossing
*y*^1^, w* ; + ; +	Derived from crossing
*y*^1^, w* ; + ; P{hs-nej+}1	Derived from crossing
*y*^1^, *nej*^Q7^, *v*^1^, *f*^1^/FM7c ; + ; +	Derived from crossing
w*/FM7c ; + ; +	Derived from crossing
*y*^1^, w* ; + ; P{hs-nej+}1, Mi{MIC}*slo*^MI02233^	Derived from crossing

^a^BDSC—obtained from the Bloomington *Drosophila* Stock Center at Indiana University (stock number in parenthesis). ^b^Aggarwal and Calvi (2004). All entries are standard *Drosophila* gene or transposon symbols. Complete description of each can be found within Flybase (http://flybase.org/) (Gramates et al., 2017) or (http://flystocks.bio.indiana.edu/). *nej*^3^, and *nej*^Q7^ are hypomorphic alleles of the *nejire* gene. FM7c, FM6 and TM3, are standard balancer chromosomes that facilitate the maintenance and manipulation of mutant alleles and transgenes carried on the opposing chromosome homolog. w*, *y*^1^, *v*^1^ and *f*^1^ are other incidental mutations known to be on the chromosome in question. P symbolizes a P element transposon that carries material enclosed within the brackets. MiMIC is a symbol representing a Minos transposon inserted into the *slo* gene. The generation of the *slo*^UAS-6b-L^ and *slo*^6b-L^ alleles are described in the “Materials and Methods” Section. Dp(1:Y)FF1, and C(1)DX are rearranged chromosomes that facilitate maintenance of the *nej*^Q7^ bearing chromosome until its use.

### Alcohol Tolerance and Resistance Assays

For all alcohol tolerance assays a population of age matched female flies is subjected to a 2-day alcohol treatment paradigm as previously described (Ghezzi et al., 2013). For all assays, 5–7 day old age-matched females were collected and sorted into replicate vials under light CO_2_ anesthesia at least 3 days before the assay. On the first day, the population is divided into two groups. One group (experimental) is exposed to alcohol vapor until sedated and then switched to fresh air for recovery. The second group (control) is left untreated. On the second day both groups are treated with the alcohol vapor again until sedation and switched to fresh air for recovery. This time however, the time of recovery is monitored and compared between the groups. If the experimental group recovers faster than the control group, the *Drosophila* strain is said to be capable of acquiring tolerance. For benzyl alcohol tolerance, each group of flies (experimental and control) consisted of three vials with 12 flies each. For the first day exposure, flies from each vial of the experimental group were sedated using a custom built benzyl alcohol vapor chamber for 15 min, while the control group was mock sedated. After sedation, the animals recovered in food vials for 24 h. On the second day, both groups were sedated in tandem using the same benzyl alcohol vapor chambers. Immediately upon sedation, flies were transferred to small plastic Petri dishes and recovery was monitored every 5 min. Flies were said to have recovered from sedation once they regain postural control. Recovery scores for each vial were plotted as the percentage of flies recovered from sedation over time. For ethanol, each group consisted of six vials with 10 flies each. On the first day, the experimental group was sedated using an ethanol-saturated air stream, while the control group was mock sedated. After sedation, the animals were allowed to recover in a fresh air environment and then returned to food vials for 24 h. On the second day, both groups were sedated in tandem using the same ethanol-saturated air stream method. Again, after sedation, the ethanol vapor was replaced with fresh air, and their recovery period monitored. Flies were said to have recovered from sedation once they regain postural control. Sedation recovery was quantified by counting the number of flies recovered from sedation in each vial at 3-min intervals. Recovery scores for each vial were plotted as the percentage of flies recovered from sedation over time. The magnitude of tolerance (i.e., the change in recovery time between experimental and control groups) was determined for each strain from the average wake-up time (AWT) of individual flies in each group and expressed as the ratio of the difference between experimental and control groups over controls:

$$\text{Tolerance Index}\;\text{=}\;\frac{{\text{AWT}}_{\left[\text{control}\right]}-{\text{AWT}}_{\left[\text{experimental}\right]}}{{\text{AWT}}_{\left[\text{control}\right]}}$$

Statistical significance was determined using Student’s *t*-test for single comparisons, or one-way analysis of variance (ANOVA) followed by Dunnett’s multiple-comparison *post hoc* test for multiple comparisons. In all assays, flies were exposed to similar amounts of alcohol in every trial. There is a slight difference in the time to sedation between ethanol and benzyl alcohol. This is mainly due to differences in delivery method. All assays were performed within a 4-h window around mid-day relative to the light cycle—between 12-noon and 4 pm of a 8 am–8 pm light cycle.

### Generation of Transgenic *slo*^UAS-6b-L^ Flies

**Figure 1:**
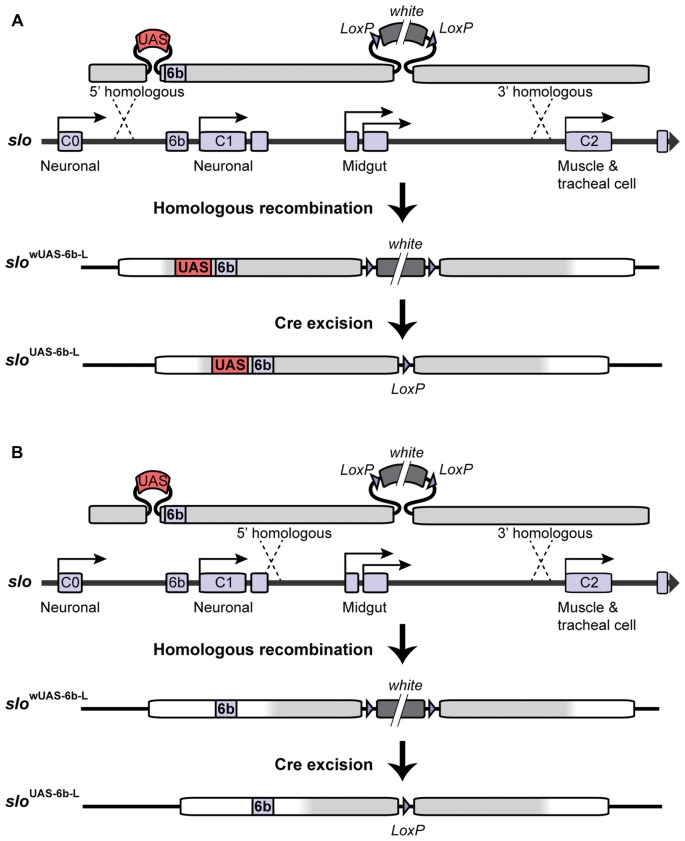
**Construction of the *slo*^UAS-6b-L^ allele.** Homologous recombination was used to construct the *slo*^UAS-6b-L^ allele. **(A)** Schematic representation of the inserted recombination construct is depicted above the *slo* transcriptional control region. **(A)** Recombination to the left of the 6b element inserts the Upstream Activating Sequence (UAS) sequence immediately 5′ of the 6b element and inserts a floxed mini-*white* gene 3′ of the 6b element. Cre recombination was used to excise the mini-*white* gene to produce the *slo*^UAS-6b-L^ allele. **(B)** Construction of the *slo*^6b-L^ control line. Recombination 3′ of element 6b inserts only the floxed mini-*white* gene. Cre recombination removes the mini-*white* gene to produce the *slo*^6b-L^ control allele of the *slo* gene.

In order to target site-specific deacetylation of the *slo* promoter, we first inserted a copy of the Upstream Activating Sequence (UAS) in the endogenous *slo* loci creating a new allele named *slo*^UAS-6b-L^. The UAS sequence is a yeast enhancer that is uniquely recognized by the binding domain of the yeast transcription factor Gal4. Addition of this sequence within the endogenous transcriptional control region of the *slo* gene allows for the targeted binding of fusion proteins containing the Gal4 DNA binding domain (Gal4^DBD^; see “Targeted Deacetylation of *slo* Promoter” Section for a description of the Gal4 construct used in this study). To generate the *slo*^UAS-6b-L^ allele, a copy of the UAS sequence was inserted at the 5′ end of the 6b DNA element in *slo* regulatory region using ends-out gene targeting strategy (Gong and Golic, 2003). At first, the 5′ homologous sequence containing a UAS next to the 6b element and the 3′ homologous sequence were amplified by PCR from Canton S genomic DNA as the template and a proofreading *PfuTurbo* DNA polymerase (Stratagene; San Diego, CA, USA). More specifically, primers 5′-GCGGCCGCACCACAAGTTCCCCAAAAC-3′ and 5′-CGTATTTAAATTCTCAGTTCTCG-3′ were used to amplify and add *Not*I and *Swa*I termini to the 5′ and 3′ end, respectively, to a 1 kb fragment upstream of the 6b element; primers 5′-TTTAAACGGAGTACTGTCCTCCGAACGGCGAGAATAGTGCTGATTTTG-3′ and 5′-TAGCTTTGTTTGCCCACGA-3′ were used to amplify and add a *Dra*I and a UAS site to the 5′ end of a 0.4 kb fragment with the 6b located at the 5′ end; primers 5′-AATTAATTACCGCGTTCGTC-3′ and 5′-ACTAGTGCATGCTCGCAAAGCAAACACACTC-3′ were used in the PCR to amplify and add the *Sph*I and the *Spe*I sites to the 3′ end of a 2.3 kb DNA fragment downstream of the 6b element. These three fragments were digested with corresponding restriction enzymes and ligated to form a 3.5 kb fragment before being inserted into the polylinker at 5′ of *white*^+^ gene marker in the ends-out vector pW25 (Gong and Golic, 2004), between the *Not*I site and the *Sph*I site. Primers 5′-GGCGCGCCATTACAAATTAACACCCAGTTGTG-3′ and 5′-CCTAGGCGAATTCGAAAAGCGTTAGC-3′ were designed to amplify and add *Asc*I terminal to the 5′ end, and add *Avr*II terminal to the 3′ end of a 3 kb DNA fragment into the polylinker to the 3′ end of *white*^+^ gene in the vector. This donor construct was introduced into the *white*^1118^ fly by standard *P* element germline transformation. All the insertion lines were mapped for the location of the donor transgene, and lines with the donor transgene on the first or the second chromosome were utilized to induce gene targeting. The target gene *slo* is located on the third chromosome. Gene targeting and the removal of the floxed mini-*white* gene proceeded as described in Gong and Golic (2003) and Li et al. (2013). Homologous recombination to one side of the 6b DNA element produced the *slo*^UAS-6b-L^ allele while recombination on the other side of the 6b element produced the matched control line *slo*^6b-L^ (see Figure 1). Therefore the control lines are the products of the same manipulations as the experimental line UAS knock-in lines and differ only in the absence of the UAS motif. Constructs were confirmed by Southern blotting, allele-specific PCR, and DNA sequencing.

### Targeted Deacetylation of *slo* Promoter

A transgenic Gal4 DNA binding domain (Gal4^DBD^) fusion line, in which the Gal4^DBD^ was fused to the catalytic domain of the histone deacetylase (HDAC) Rpd3 was used to induce targeted deacetylation of the UAS-tagged *slo* promoter. The fusion transgene is controlled by a heat-inducible promoter (Hsp70). The *slo*^6b-UAS-L^ line, and the corresponding *slo*^6b-L^ control line, were crossed to the Hsp70-Gal4^DBD^:Rpd3/FM6 line (Aggarwal and Calvi, 2004) to create the stable Hsp70-Gal4^DBD^:Rpd3/FM6; + ; *slo*^UAS-6b-L^ and Hsp70-Gal4^DBD^:Rpd3/FM6; + ; *slo*^6b-L^ lines. A 1-h heat-shock (HS) was used to induce the Gal4^DBD^:Rpd3 fusion protein in these lines 30 min after the first alcohol treatment. The Gal4^DBD^:Rpd3 fusion protein will only bind and deacetylate the *slo* 6b region in the presence of the UAS sequence.

### Analysis of CBP modENCODE Data

To analyze the baseline profile of CBP binding of target genes, genome-wide ChIP-seq data of CBP binding was obtained from the National Human Genome Research Institute model organism ENCyclopedia Of DNA Elements (modENCODE) database. This data was collected by the laboratory of Kevin P. White at the University of Chicago from *Drosophila* Adult Female 2–3 days old and has been made available through the modENCODE database (accession#: modEncode_863) or the NCBI GEO public data repository (accession# GSM408982). Genome-wide binding data was visualized using the Integrated Genome Browser (Freese et al., 2016) and the binding landscapes for six different genes exported for presentation.

### Chromatin Immunoprecipitation and qPCR of H4ac-Enriched Regions

To measure the effects of the *nej* mutation on the alcohol-induced histone H4 acetylation pattern, changes in H4 acetylation were quantified using ChIP-qPCR as described in Ghezzi et al. (2013). All ChIP experiments were performed following the modENCODE consortium guidelines (Landt et al., 2012). For ethanol, approximately 300 flies were housed in a perforated 500 ml plastic bottle chamber. Humidified air saturated with ethanol vapor was delivered to flies in the chamber using an ethanol vapor inebriator set to 15 ml air per minute. For the untreated control, ethanol free humidified air was delivered to the chamber. Flies were placed in each chamber and exposed until the ethanol group was completely sedated (~15 min). For benzyl alcohol, a similar number of flies were placed in a 200 ml glass tube coated with benzyl alcohol. For the untreated control, a similar benzyl alcohol-free tube was prepared. Flies were placed in each tube and exposed until the benzyl alcohol group was completely sedated (~15 min). All flies were then transferred to fresh-food bottles for recovery. Six hours after treatment, chromatin was isolated from *Drosophila* heads from wild type (CS) and mutant (w*, *nej*^3^/FM7c). Flies were frozen in liquid nitrogen, vortex decapitated and the heads were collected by sieving. Heads were homogenized and cross-linked with 2% formaldehyde for 2 min. Solubilized chromatin was sonicated on ice 6 × 30 s, to produce fragments of approximately 200–1000 bp as described by Wang et al. (2007). DNA associated with acetylated histone H4 was captured using a 1:200 dilution of ChIP anti-acetyl-Histone H4 Antibody (Rabbit Polyclonal Antibody) from EMD Millipore (catalog # 06–866; Billerica, MA, USA) and the immunocomplexes recovered using Pierce^TM^ Protein A Agarose beads (Thermo Fisher Scientific, catalog # 20333; Waltham, MA, USA). A 10% aliquot from each chromatin sample was held back to serve as input and was thus not subjected to immunoprecipitation. After washes and finally elution, the DNA corresponding to the immunoprecipitated and input material was extracted and purified. Quantitative PCR was used to assay 6 conserved DNA elements within the *slo* transcriptional control region: C0, 6b, C1, cre1, 55b and cre2, and the internal control *Gpdh*. The primers used were: C0 (5′-ATCGAACGAAGCGTCCAG-3′, 5′-CGACGCGCTCAAACG-3′), 6b (5′-CCAGCAGCAATTGTGAGAAA-3′, 5′-CGAAGCAGACTTGAAAGCAA-3′), C1 (5′-ACAAACCAAAACGCACAATG-3′, 5′-AATGGATGAAGGACTGGGAGT-3′), cre1 (5′-GATGGGAAAGCGAAAAGACAT-3′, 5′-CATGTCCGTCAAAGCGAAAC-3′), 55b (5′-ACCCAATTGAATTCGCCTTGTCTT-3′, 5′-CCCACTCTCCGGCCATCTCT-3′), cre2 (5′-TGGATTGCGACCGAGTGTCT-3′, 5′-ATCAATACGATAACTGGCGGAAACA-3′), and *Gpdh* (5′-GCATACCTTGATCTTGGCCGT-3′, 5′-GCCCTGAAAAGTGCAAGAAG-3′). The relative amount of the acetylated H4 histone was calculated by the ΔΔCT method. Fold enrichment over control is equal to 2∧(Ct^Input^ − Ct^IP^)_experimental_/2∧(Ct^Input^ − Ct^IP^)_control_. All data were normalized to the *Gpdh* values. Chromatin immunoprecipitation assays were performed at least three times from independent chromatin samples and the mean and SEM were calculated. Statistical significant changes between wild-type and mutant were determined by two-way ANOVA.

### Measurement of Gene-Expression by RT-qPCR

For quantification of gene expression, total RNA was extracted from heads of age-matched female flies (~75 heads per replicate), 6 h after treatment with either ethanol, benzyl alcohol, 1 h HS, or untreated controls. Extraction was performed using a single-step RNA isolation protocol (Ausubel, 1994). RNA was treated with RNase free DNase I (Ambion, Austin, TX, USA), purified by acid phenol/chloroform extraction (Ambion, Austin, TX, USA) and precipitated with ethanol. Reverse transcription was performed from 50 ng of total RNA using the SuperScript VILO cDNA Synthesis Kit (Invitrogen/Life technologies, Carlsbad, CA, USA), and amplified using the SYBR Green PCR Master Mix (Applied Biosystems/Life technologies, Carlsbad, CA, USA) in a ViiA 7 Real-time PCR system (Applied Biosystems, Carlsbad, CA, USA). Quantification of mRNA for each gene was determined relative to the *Cyp1* mRNA using the ΔΔCT method. Primer sequences are as follows: *slo* (5′-AAACAAAGCTAAATAAGTTGTGAAAGGA-3′ and 5′-GATAGTTGTTCGTTCTTTTGAATTTGA-3′); *para* (5′-GAGCCCCAAGTACTATTTCCAG-3′ and 5′-GTCCCAGTTCCAATAGCGATAG-3′); *eag* (5′-GTATCGGTTCCCTGTTCAGTG-3′ and 5′-CCAGGTAGCGATCCAGTTTTC-3′); *brp* (5′-CGAGAAGCTGGACAAGACG-3′ and 5′-CGAATGACTCCGACTCGTATTG-3′); *Teh2* (5′-CTCGTGGGAGAACAATCTGTAC-3′ and 5′-CAGTACCAATAGCTGAGCACC-3′); *pum* (5′-GCCCAGATGCCGTACTATG-3′ and 5′-CGTTCCCTGTTGCGGAATC-3′); *nej* (5′-AGAAGGAGTTTATGGATGACAGC-3′ and 5′-GTTCACATTCTTGCCCTTGC-3′); *Cyp1* (5′-GAGAAGGGATTCGGGTACAAG-3′ and 5′-TGTTGCCGTAGATGGACTTG-3′). A minimum of five replicate RT-PCR reactions was performed from independent RNA samples. Statistical significance was calculated using the One-way ANOVA for each gene with Dunnett’s *post hoc* test for comparisons to the untreated controls.

All qPCR measurements were made in accordance to MIQE standards (Bustin et al., 2009). The genes used for normalization have previously been shown to not be affected by the treatments described here (Wang et al., 2007). In addition, when normalized against total input RNA or input chromatin conceptually identical results were obtained.

## Results

### Targeted Deacetylation of the *slo* Promoter Attenuates Alcohol Tolerance

Ethanol and benzyl alcohol produce mutual cross-tolerance indicating that the underlying mechanism of tolerance must be the same for these two alcohols. In Ghezzi et al. (2013) we screened for genes involved in producing alcohol tolerance by identifying genes whose histone acetylation status increased following both a single ethanol sedation and following a single benzyl alcohol sedation. Mutant, RNAi and overexpression analysis showed that ~80% of such genes were involved in the capacity to acquire tolerance to these drugs. In the previous work we assumed that the correlation between histone acetylation, alcohol induction and tolerance reflected a functional relationship. To confirm this relationship, here we specifically antagonized alcohol-induced histone acetylation of an alcohol tolerance gene by positioning a deacetylase in its transcriptional control region and then asking whether suppressing histone acetylation interfered with gene induction and alcohol tolerance.

To accomplish this, we used a modified Gal4/UAS system. The UAS sequence is recognized by the DNA-binding domain of the yeast Gal4 transcription factor and has been extensively used to manipulate gene expression in *Drosophila* (Brand and Perrimon, 1993). For this study, we generated a UAS/Gal4 bipartite system comprised of: (1) a transgenic heat-inducible fusion protein consisting of the DNA-binding domain of Gal4 (Gal4^DBD^) and the HDAC catalytic domain of the *Drosophila* RPD3; and (2) a Gal4 responsive UAS site, which was engineered into the endogenous *slo* gene. In this system, when induced by a brief HS, the HDAC catalytic domain of RPD3 can be directed specifically to the *slo* promoter as it specifically recognizes the inserted UAS sequence. Localization of Gal4^DBD^:RPD3 at this position would be expected to remove histone acetylation marks within the *slo* promoter region.

**Figure 2:**
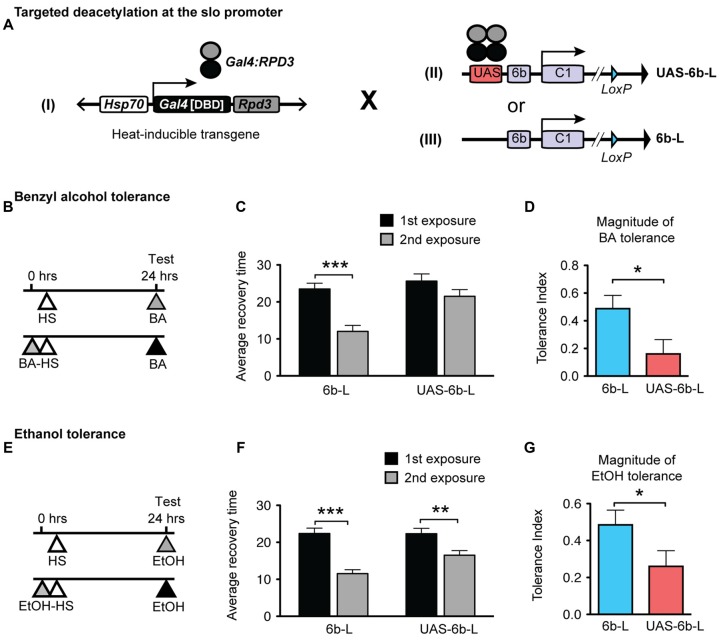
**Targeted deacetylation of the *slo* promoter can interfere with alcohol tolerance. (A)** Schematic depiction of the genetic cross used to inducibly tether the RPD3 HDAC activity within the *slo* transcriptional control region: transgenic flies that carry a heat-inducible *Gal4*^DBD^:*Rpd3* cDNA (I) were crossed to flies carrying either the *slo*^UAS-6b-L^ allele (II) or to flies carrying the *slo*^6b-L^ allele that lacks the UAS site (III). **(B,E)** Paradigm to test for an effect of the tethered RPD3 on alcohol tolerance. Animals were sedated with ethanol **(B)** or benzyl alcohol **(E)** once (top line) or twice (bottom line; 24 h between sedations). All animals were heat treated to activate *Gal4*^DBD^:*Rpd3* expression. The time of heat activation was 30 min after the first alcohol treatment. **(C,F)** Average recovery time of animals after a single (black bar) or after two consecutive (gray bar) sedations with benzyl alcohol **(C)** or ethanol **(F)**. See Supplementary Figure S2 for the corresponding recovery curves. Error bars represent SEM (Student’s *t*-test: *** in **C** denotes *P* < 0.0001, *n* = 32 [2nd exp], 30 [1st exp.]; ** in **F** denotes *P* = 0.0026, *n* = 54 [2nd exp], 43 [1st exp.]; *** in **F** denotes *P* < 0.0001, *n* = 47 [2nd exp], 47 [1st exp.];). **(D,G)** Magnitude of tolerance induced in the *Hsp-Gal4*^DBD^:*Rpd3*/FM6 ; ; *slo*^6b-L^ animals (6b-L) and in the *Hsp-Gal4*^DBD^:*Rpd3*/FM6 ; ; *slo*^UAS-6b-L^ (UAS-6b-L) animals. Error bars represent SEM (Student’s *t*-test: * in **D** denotes *P* = 0.0238, *n* = 32 [6b-L], 33 [UAS-6b-L]; * in **G** denotes *P* < 0.0367, *n* = 47 [6b-L], 43 [UAS-6b-L]).

Insertion of the UAS DNA element into the *slo* transcriptional control region was performed by the homologous recombination method (see Figure 1 and Gong and Golic, 2003). The UAS was positioned adjacent to the so-called 6b DNA element, between the two neural promoters, which are induced by alcohol sedation (Ghezzi et al., 2004; Cowmeadow et al., 2006). The *slo* gene and the 6b element have been implicated in the alcohol tolerance response (Li et al., 2013; Krishnan et al., 2016). Downstream of the 6b element there also remains a single LoxP site that is a remnant of the construction method. This new *slo* allele is called *slo*^UAS-6b-L^. At the same time, the homologous recombination process generated the control allele. This control allele carries the downstream LoxP site but does not carry a UAS site. The control allele is called *slo*^6b-L^. These modifications, by themselves, do not produce an obvious behavioral phenotype. The second part is the heat-inducible Hsp70 promoter that drives the expression of an artificial transcription factor consisting of the DNA-binding domain of the yeast Gal4 transcription factor (Gal4^DBD^) and the HDAC catalytic domain of the *Drosophila* RPD3 (generated by Aggarwal and Calvi, 2004). Like all HDACs, RPD3 has relaxed substrate specificity and is thought to remove all or most types of histone acetylation, although for each modification the removal rate may differ (Feller et al., 2015). The genetic assembly of both parts of this UAS/Gal4 bipartite system is depicted in Figure 2A. The final genotypes of the tested lines were *Hsp-Gal4*^DBD^:*Rpd3*/FM6 ; ; *slo*^UAS-6b-L^ for the experimental line, and *Hsp-Gal4*^DBD^:*Rpd3*/FM6 ; ; *slo*^6b-L^ for the control line.

The tolerance assay used to probe the consequences of positioning the Gal4:RPD3 fusion protein within the *slo* promoter region is a recovery-from-sedation assay in which tolerance is induced and measured in a 2-day paradigm (originally described in Ghezzi et al., 2004; Cowmeadow et al., 2005). On day 1, one group of age- and sex-matched flies are sedated with alcohol vapor (experimental group) while the second group is not exposed to alcohol vapor (control group). Twenty-four hours later, both groups are sedated with alcohol vapor in tandem, switched to a fresh air environment for recovery, and the time to recovery from sedation compared. If the experimental group recovers faster than the control group, the fly line is said to be capable of acquiring tolerance. When the *Hsp70*-*Gal4*:*Rpd3* transgene is not induced, the test animals acquire tolerance to both benzyl alcohol and to ethanol (Supplementary Figure S1). To induce the *Hsp70*-*Gal4*:*Rpd3* transgene, both the experimental and control groups are subjected to a 30 min 37°C heat pulse (at all other times the flies are maintained at ~22°C). A schematic of the tolerance assay paradigm is shown in Figure 2B (for benzyl alcohol) and Figure 2E (for ethanol). Activation of the Gal4:RPD3 transgene in animals with an insertion of a UAS element within the *slo* promoter region (*slo*^UAS-6b-L^) interfered with the capacity to acquire both benzyl alcohol and ethanol tolerance (6b-L-UAS; Figures 2C,F, respectively). In these animals, activation of the transgene blocked the acquisition of benzyl alcohol tolerance completely and reduced the magnitude of tolerance produced to sedation with ethanol vapor. The tolerance index—the difference in recovery time between exposures— is significantly reduced in the UAS lines for both drugs (Figure 2D for benzyl alcohol, and Figure 2G for ethanol). This does not appear to be a product of a nonspecific effect of Gal4:RPD3 expression, because activation of the Gal4:RPD3 transgene in control animals lacking the UAS element (*slo*^6b-L^) did not interfere with the acquisition of tolerance to either benzyl alcohol or to ethanol (6b-L; Figures 2C,F, respectively).

### The Histone Acetyltransferase CBP Binds to a Network of Alcohol-Responsive Genes

During gene activation histone acetyltransferases are recruited to promoter regions by transcription factors. In Ghezzi et al. (2013), alcohol-induced histone acetylation was used in a genomic screen for alcohol tolerance genes. One of the induced genes was *nejire*, which encodes the *Drosophila* homolog to CBP/p300. This transcription cofactor is known to be recruited by the CREB transcription factor which has been previously shown to be involved in the alcohol-related induction of *slo* gene expression and in the production of tolerance to benzyl alcohol (Wang et al., 2007, 2009). Thus, CBP is a strong candidate for producing the histone acetylation involved in alcohol-induced activation of the *slo* gene and perhaps other alcohol response genes.

**Figure 3:**
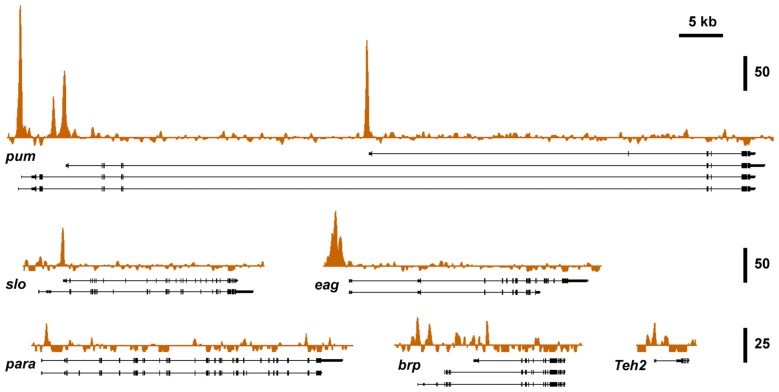
**Nejire/CBP binding at different tolerance genes.** A survey of basal CBP binding across the six alcohol tolerance genes was acquired from the modENCODE database (Celniker et al., 2009). Peak plots depict ChIP-seq signal obtained from immunoprecipitated chromatin from adult female flies using a CBP antibody superimposed on representative Refseq transcript isoforms for six known tolerance genes. All of these genes have been previously shown to be induced by both benzyl alcohol and ethanol (Ghezzi et al., 2013). Strong Nejire/CBP binding is localized near the transcription start sites or within the first exon of these genes. Vertical scale bars denote fold enrichment over input.

To investigate if known alcohol responsive genes can be regulated by CBP, we obtained CBP ChIP-seq data from the modENCODE project directed by Kevin White, which is aimed at mapping the association of transcription factors on the genome of *Drosophila* (Celniker et al., 2009; Nègre et al., 2011). Figure 3 shows that *slo* and five of the other alcohol tolerance genes described in Ghezzi et al. (2013) have CBP protein bound at or near the respective transcription start sites. The genes analyzed here belong to an interrelated network of genes with a direct role in producing alcohol tolerance. All genes in this network were previously shown to display similar histone H4 acetylation changes after alcohol exposure; and have been validated through a mutant screen to play a critical role in the development of alcohol tolerance (Ghezzi et al., 2013). Gene ontology analysis indicates that this set of genes fall into important interconnected categories and encode a set of proteins that are tightly associated with the regulation of synaptic plasticity. The genes are: *slo, a* BK-type Ca2^+^-activated K^+^ channel (Atkinson et al., 1991); *eag*, a voltage-gated K^+^ channel gene (Brüggemann et al., 1993); *Teh2*, an ion-channel β subunit (Derst et al., 2006); the synaptic active zone component, *brp* (Kittel et al., 2006); the voltage-gated Na^+^ channel gene, *para* (Loughney et al., 1989); and the activity-dependent translational repressor known to regulate synaptic proteins, *pum* (Mee et al., 2004). All of these genes showed changes in expression in response to sedation to both benzyl alcohol and ethanol (Ghezzi et al., 2013). This, alone however, does not mean that the CBP transcription cofactor is involved in changes in gene expression that contribute to an alcohol-induced behavior.

### Mutations in *nejire* Suppress Alcohol Tolerance

**Figure 4:**
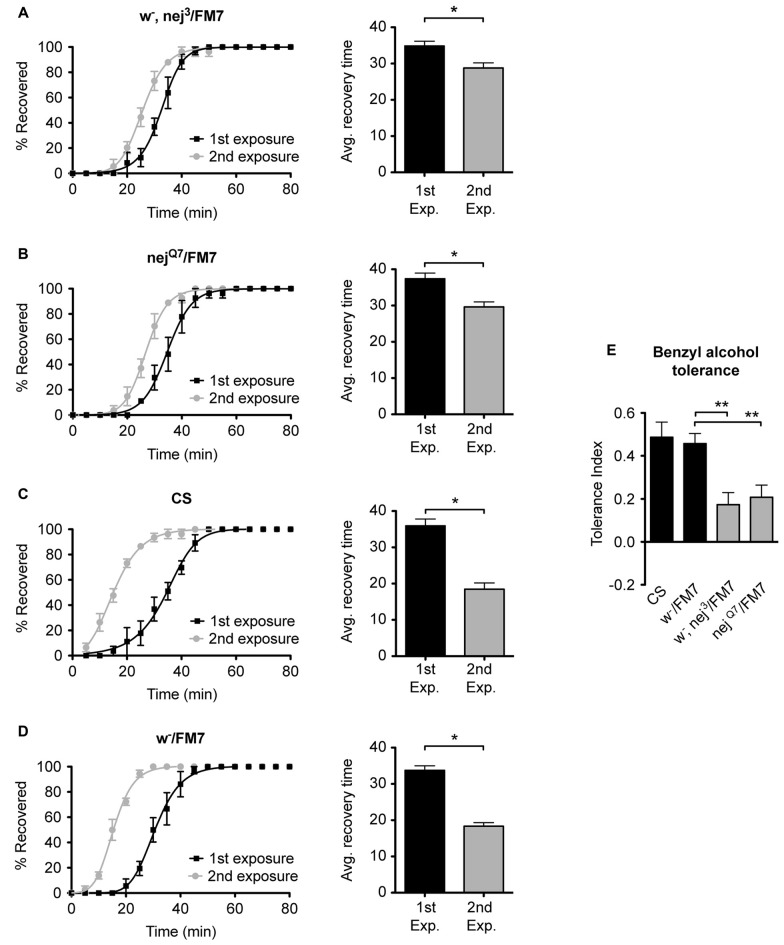
**Mutations in the *nejire* gene reduce the capacity for benzyl alcohol tolerance.** Recovery curves from benzyl alcohol sedation, and the respective average recovery times, for *nej*^3^ heterozygous flies **(A)**, *nej*^Q7^ heterozygous flies **(B)**, the wild type (WT) Canton-S **(C)** and the *w*-/FM7 background control flies **(D)**. In each recovery curve graph, the black curve represents recovery from a first benzyl alcohol treatment (1st exposure), whereas the gray curve represents recovery from a second benzyl alcohol treatment (2nd exposure). For each fly strain, the average recovery time of animals after the first benzyl alcohol treatment (1st exp.) or after a second benzyl alcohol treatment (2nd exp.) are shown to the right of each panel. Error bars represent SEM (Student’s *t*-test: *denotes *P* < 0.05, *n* > 27). The difference in recovery times for all strains is depicted as the tolerance index **(E)** for the heterozygous *nej*^3^ or *nej*^Q7^ mutant alleles in comparison to the Canton S or *w*-/FM7 control flies. Error bars represent SEM (One-way analysis of variance (ANOVA) w/Dunnett post test: **denotes *P* < 0.01; *n* = 36 [*w*-/FM7], 31 [*w*-, *nej*^3^/FM7], 27 [*nej*^Q7^/FM7], 27 [CS]).

**Figure 5:**
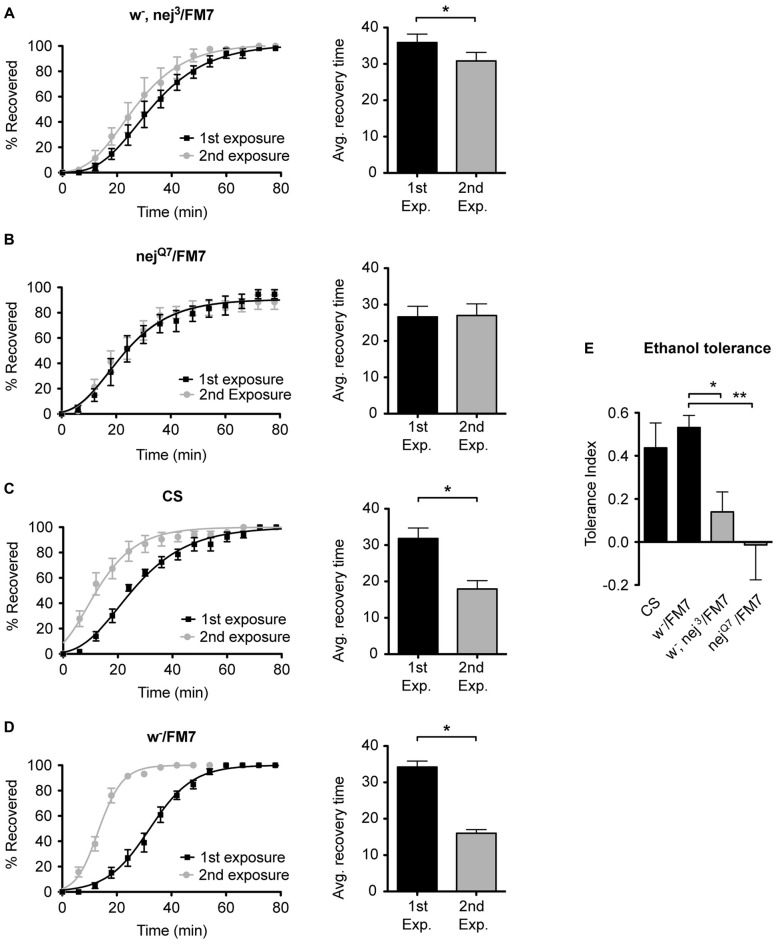
**Mutations in the *nejire* gene reduce the capacity for ethanol tolerance.** Recovery curves from ethanol sedation, and the respective average recovery times, for *nej*^3^ heterozygous flies **(A)** and *nej*^Q7^ heterozygous flies **(B)**, the WT Canton-S **(C)** and the *w*-/FM7 background control flies **(D)**. In each recovery curve graph, the black curve represents recovery from a first ethanol treatment (1st exposure), whereas the gray curve represents recovery from a second ethanol treatment (2nd exposure). For each fly strain, the average recovery time of animals after the first ethanol treatment (1st exp.) or after a second ethanol treatment (2nd exp.) are shown to the right of each panel. Error bars represent SEM (Student’s *t*-test: *denotes *P* < 0.05, *n* > 31). The difference in recovery times for all strains is depicted as the tolerance index **(E)** for the heterozygous *nej*^3^ or *nej*^Q7^ mutant alleles in comparison to the Canton S or *w*-/FM7 control flies. Error bars represent SEM (One-way ANOVA w/Dunnett post test: *denotes *P* < 0.05; **denotes *P* < 0.01; *n* = 58 [*w*-/FM7], 48 [*w*-, *nej*^3^/FM7], 31 [*nej*^Q7^/FM7], 51 [CS]).

To examine the role of the histone acetyltransferase CBP in the induction of alcohol tolerance we examined two different mutant alleles of the *nejire* gene—*nej*^3^ and *nej*^Q7^. The recessive lethal *nej*^3^ mutant allele carries a 2–3 kb deletion near the 5′ end of the gene and appears to be a null mutation (Akimaru et al., 1997). Although the *nej*^3^ heterozygous flies develop normally, they show signs of reduced CBP activity, as demonstrated by the enhancement of hypomorphic phenotype of one of CBP’s transcriptional co-activators—*dpp* (Waltzer and Bienz, 1999). We observed that *nej*^3^ heterozygous animals also had a greatly diminished capacity for the acquisition of both benzyl alcohol and ethanol tolerance, as shown by the relatively small shift in recovery times between the first and second exposures (Figures 4A, 5A, respectively). Similarly, the second *nejire* mutation tested—the *nej*^Q7^ allele—also interferes with tolerance. The *nej*^Q7^ allele is a strong antimorphic allele (Florence and McGinnis, 1998). As with the *nej*^3^ mutants, *nej*^Q7^ heterozygous flies also develop normally but show a greatly diminished capacity for the acquisition of both benzyl alcohol and ethanol tolerance (Figures 4B, 5B, respectively). This is in contrast with the robust tolerance displayed by the wild-type strain CS (Figures 4C, 5C, for benzyl alcohol and ethanol respectively). As tested, the *nejire* mutant stocks are also mutant for the *white* (*w*) gene and carry an FM7 balancer chromosome. However, the *w-/FM7* combination does not appear to contribute to the abnormal tolerance phenotype since the control *w-*/FM7 line shows the same magnitude of tolerance as do wild type (WT) animals (Figures 4D, 5D, for benzyl alcohol and ethanol respectively). In summary, tolerance in both *nejire* mutants is significantly reduced as compared to that of the appropriate background controls. This is shown as a reduced tolerance index to benzyl alcohol and ethanol in Figures 4E, 5E, respectively.

### Induction of *nejire* Expression Phenocopies Tolerance

**Figure 6:**
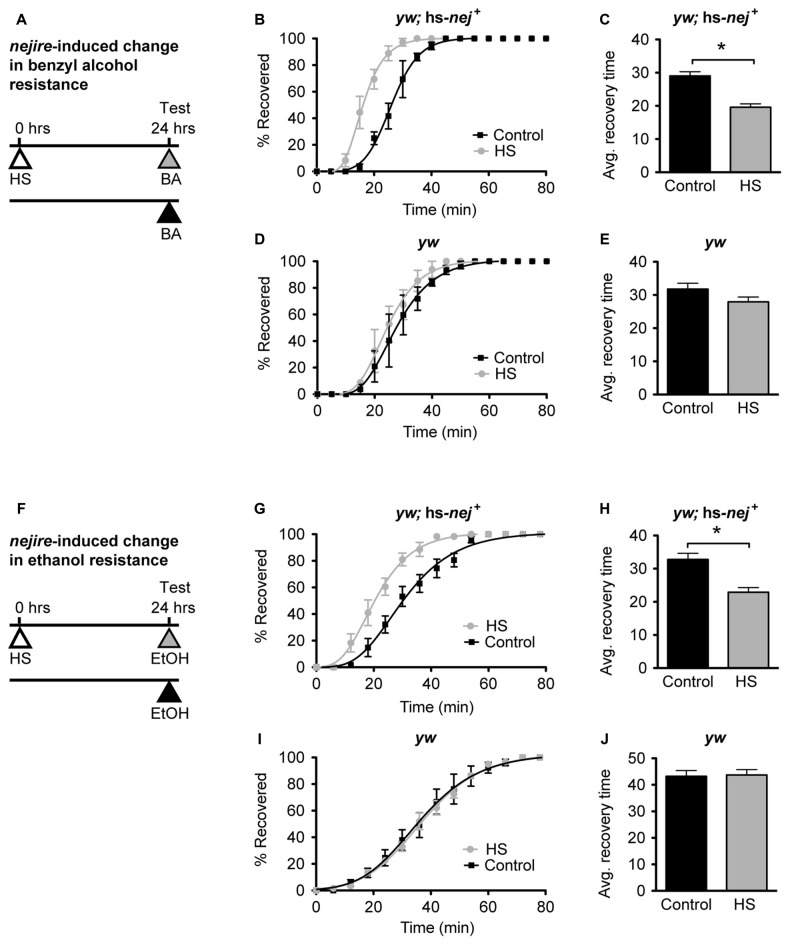
***nejire* induction produces alcohol resistance. (A,F)** Schematic depiction of the 2-day treatment protocol. A heat-shock (HS) inducible transgenic construct in which the *Hsp70* promoter drives expression of a *nejire* cDNA was used to drive expression of CBP, 24 h prior to sedation with either benzyl alcohol (top, **A–E**) or ethanol (bottom, **F–J**). **(B,C)** Recovery curves **(B)** and average-recovery time **(C)** from benzyl alcohol sedation of transgenic *yw*; hs-*nej*^ +^ flies in which CBP was induced (HS) or uninduced (Control). Error bars represent SEM (Student’s *t*-test: *denotes *P* < 0.0001, *n* = 52 [Control], 54 [HS]). **(D,E)** Recovery curves **(D)** and average-recovery time **(E)** from benzyl alcohol sedation of control *yw* flies lacking the hs-*nej*^ +^ transgene in which a HS or control treatment (Control) was applied. Error bars represent SEM (Student’s *t*-test: not significant). **(G,H)** Recovery curves **(G)** and average-recovery time **(H)** from ethanol sedation of transgenic *yw*; hs-*nej*^ +^ flies in which CBP was induced (HS) or uninduced (Control). Error bars represent SEM (Student’s *t*-test: *denotes *P* < 0.0001, *n* = 36 [Control], 36 [HS]). **(I,J)** Recovery curves **(I)** and average-recovery time **(J)** from ethanol sedation of control *yw* flies lacking the hs-*nej*^ +^ transgene in which a HS or control treatment (Control) was applied. Error bars represent SEM (Student’s *t*-test: not significant).

In Ghezzi et al. (2013) ethanol and benzyl alcohol treatments that induce tolerance were shown to also induce *nejire* gene expression in fly heads. Alcohol tolerance is defined as an alcohol-induced increase in alcohol resistance, whereas resistance refers to the relative level of response to alcohol in alcohol-naive animals. To determine whether increased *nejire* expression, by itself, phenocopies alcohol tolerance, we used an inducible transgene to manipulate *nejire* expression, and measured the shift in resistance. In the hs-*nej*^ +^ transgene, a heat-inducible Hsp70 promoter drives expression of a *nejire* cDNA (Akimaru et al., 1997; Attrill et al., 2016). In the experiment presented in Figure 6, one group of flies is heat treated on day 1 to boost expression of the hs-*nej*^+^ transgene while the other set of flies is not. Then on day 2 both groups are ethanol-sedated in tandem and the recovery curves compared. Compared to a typical tolerance test, in this experiment, the first day exposure to alcohol vapor has been replaced by the heat induction of the *nejire* transgene. This way we can directly test if an increase in *nej* expression can elicit the change in resistance that produces tolerance. The 2-day protocol is depicted in Figures 6A,F for benzyl alcohol and ethanol respectively.

In this paradigm, a 1-h HS (37°C) was used to induce the transgene 24 h before sedation with either alcohol. This treatment produces robust induction of *nejire* mRNA (Supplementary Figure S3). On the alcohol treatment day, the induced animals, and the appropriate uninduced controls, were treated with benzyl alcohol or ethanol vapor until sedated, and the recovery time in a fresh-air environment was monitored. For both alcohols, the induced flies recovered faster from sedation than their uninduced counterparts as depicted by the recovery curves (Figures 6B,G). A leftward shift in the recovery curves indicates increased resistance. Similarly, the average recovery time of *nejire*-induced flies is significantly shorter than that of the uninduced controls (Figures 6C,H). This effect cannot be attributed to idiosyncratic side effects of the HS treatment, because flies that do not carry the heat-inducible transgene show no change in resistance to either alcohol as shown for the *yw* background control stock (Figures 6D,E, for benzyl alcohol; Figures 6I,J for ethanol) and as reported in Ghezzi et al. (2004) and Cowmeadow et al. (2006).

### CBP Acts Upstream of *slo* to Induce Alcohol Tolerance

In Ghezzi et al. (2013) mutant or RNAi-mediated suppression of any one of the alcohol-induced genes shown in Figure 3 (except *para*, which was not confirmed because *para* suppression was lethal) have been shown to be required for the acquisition of alcohol tolerance. For some of the genes, transgenic induction also showed that induction phenocopies tolerance. The most extensively studied gene in this group has been the *slo* BK type-Ca^2+^-activated K^+^ channel gene. A mutation that impairs *slo* expression blocks tolerance to both ethanol and benzyl alcohol, whereas artificial induction of the gene, increases resistance to sedation with either alcohol (Ghezzi et al., 2004; Cowmeadow et al., 2006). Moreover, acetylation of the promoter region of the gene has been shown to be a critical step in the induction of *slo* during the development of tolerance (Wang et al., 2007). This evidence, prompted us to investigate the relationship between CBP and *slo*, and specifically, whether the Nejire/CBP histone acetyltransferase was important for the acquisition of *slo*-dependent tolerance.

**Figure 7:**
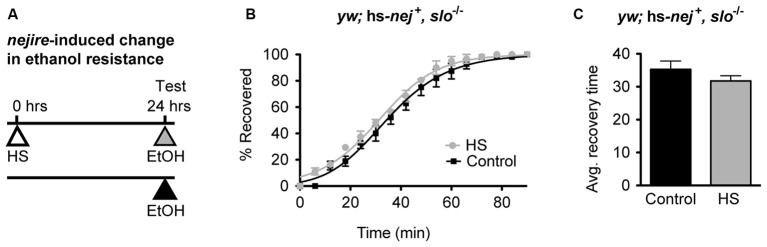
**A mutation in *slo* blocks CBP-induced alcohol resistance. (A)** Schematic depiction of the 2-day treatment protocol. A HS inducible transgenic construct in which the *Hsp70* promoter drives expression of a *nejire* cDNA was used to drive expression of CBP in a null *slo* mutant background. Induction of the transgene was performed 24 h prior to sedation with ethanol. **(B)** Ethanol recovery curves of *slo*^MI02233^ (*slo*^−/−^) flies carrying the hs-*nej*^ +^ transgene. The black curve represents recovery from uninduced flies (Control), whereas the gray curve represents recovery from a CBP-induced flies (HS). **(C)** The average recovery time of control and heat-shocked induced flies (HS) is plotted. CBP induction does not affect recovery time from ethanol. Error bars represent SEM (Student’s *t*-test: not significant).

If the induction of the Nejire/CBP protein acts upstream of *slo* in the production of alcohol tolerance then one would expect the *nejire*-induced change in resistance phenotype observed in Figure 6 to be epistatically blocked by a *slo* loss-of-function mutation. To test for such an epistatic interaction, we used the *slo*^MI02233^ mutant that expresses a truncated version of the gene. Animals homozygous for this allele display the sticky-feet phenotype characteristic of all *slo* null alleles (Elkins et al., 1986; Atkinson et al., 1991, 2000). The change in ethanol resistance of flies heterozygous for the hs-*nej*^ +^ transgene and homozygous for the *slo*^MI02233^ mutant allele was again tested using a 2 day paradigm (Figure 7A). On day 1, animals were either treated with a HS to induce the hs-*nej*^ +^ transgene, or were left untreated, while on day 2, both groups were sedated with alcohol and the change in resistance measured. The hs-*nej*^ +^ induction protocol is identical to that used in Figure 6, however in these animals, the 1-h HS (37°C) was used to induce the *nejire* transgene in the *slo*^MI02233^ genetic background. In this case, the heat-treated flies (*nejire*-induced) recovered from sedation at the same rate as their non heat-shocked (uninduced) siblings and produced overlapping recovery curves and recovery times (Figures 7B,C). This indicates that the loss-of-function *slo* allele has epistatically blocked the effect of HS activation of the *nejire* transgene, suggesting that a functional *slo* gene is necessary for the induction of tolerance by *nejire*.

### A Mutation in *nejire* Precludes Alcohol-Induced Histone H4 Acetylation of *slo*

**Figure 8:**
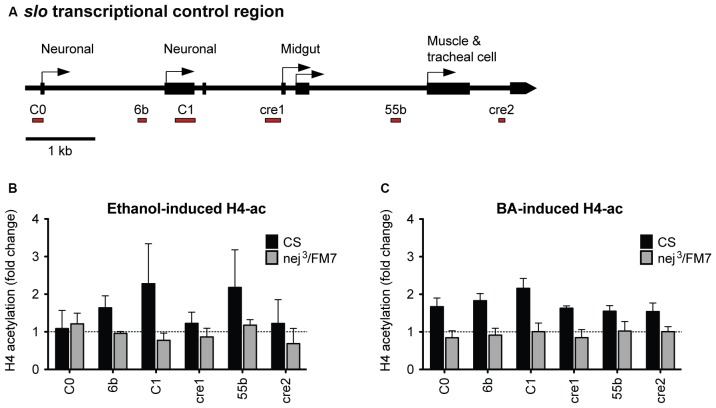
**Alcohol-induced histone H4 acetylation change across the *slo* transcriptional control region are suppressed by a mutation in the *nejire* gene. (A)** Levels of histone H4 acetylation were measured by ChIP-qPCR at six discrete highly conserved positions within the *slo* transcriptional control region. These regions are depicted below the *slo* transcriptional control region map, as red bars. **(B)** H4 acetylation changes induced by ethanol 6 h after treatment in CS and *nej*^3^/FM7 mutants. The mutation in *nejire* affects the histone acetylation pattern (two-way ANOVA, *p* = 0.04). **(C)** H4 acetylation changes induced by benzyl alcohol 6 h after treatment in CS and *nej*^3^/FM7 mutants. The mutation in *nejire* affects the histone acetylation pattern (two-way ANOVA, *p* < 0.0001).

Because a mutation in *slo* completely eliminates the increase in resistance to ethanol generated by artificial induction of *nejire*, we hypothesized that *nejire* is responsible for the acetylation of the *slo* promoter that leads to an increase in alcohol resistance. If this is true, we should observe a reduction in alcohol-induced acetylation of the *slo* transcriptional control region in the heterozygous *nej*^3^ mutants. To test this, we measured the levels of histone H4 acetylation across six distinct DNA elements within the *slo* transcriptional control region (Figure 8A). Acetylation at these sites has been previously associated with alcohol-induced *slo* gene expression and the production of tolerance to both benzyl alcohol and ethanol (Li et al., 2013; Krishnan et al., 2016). The CBP/p300 family of histone acetyltransferases have also been shown to catalyze acetylation of H2AK5, H3K14, H3K18, H3K23, H3K27, H3K64, H4K5 and H4K8 (see Tables 4 and 7 in Allis et al., 2015). Thus, measuring H4 acetylation can provide a direct measure of CBP activity. As shown in Figures 8B,C, the *nej*^3^ mutation blocks alcohol-induced acetylation across the *slo* transcriptional control region, for both ethanol and benzyl alcohol.

In wild-type animals, histone H4 acetylation is increased across the entire *slo* transcriptional control region after exposure to either alcohol, with the most prominent peaks centered on the C1 neuronal promoter region. C1 is a neural-specific promoter that is known to be transcriptionally activated after alcohol sedation (Ghezzi et al., 2004; Cowmeadow et al., 2006). In the *nej*^3^ heterozygous mutant however, the acetylation changes are completely suppressed and peaks are no longer detected. These observations demonstrate that alcohol-induced acetylation at the *slo* gene rely on a fully-functioning CBP protein system, and suggest that CBP is responsible for the acetylation events.

### A Mutation in *nejire* Blocks Induction of Alcohol-Response Genes

**Figure 9:**
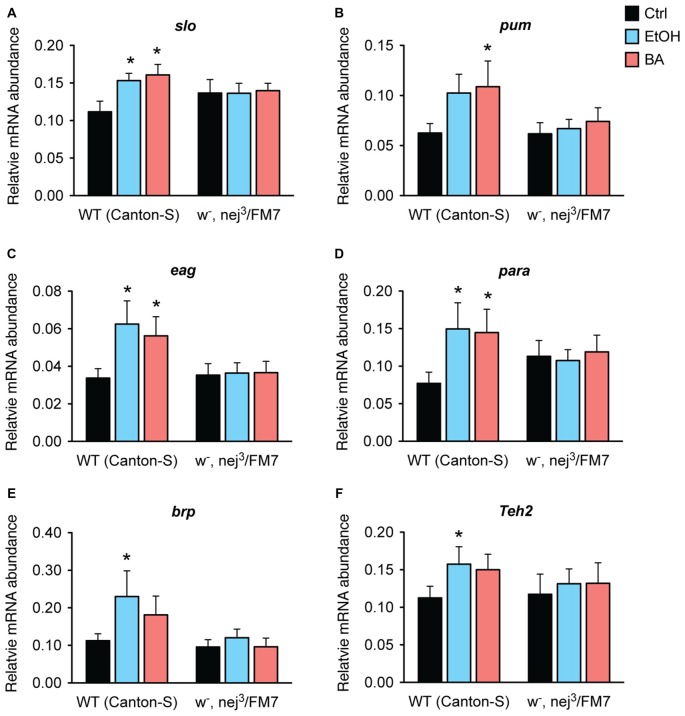
**Alcohol induction of tolerance genes is blocked by a mutation in *nejire*.** Shown is the mRNA abundance of six different alcohol-responsive genes, relative to the abundance of the internal control gene (*Cyp1*), 6 h after a sedative exposure to ethanol (light blue), benzyl alcohol (red), or in an untreated control (black) in WT or *nejire* mutants (*w*-, *nej*^3^/FM7). The genes tested were: **(A)** *slo*, **(B)** *pum*, **(C)** *eag*, **(D)** *para*, **(E)** *brp*, **(F)** *Teh2*. Error bars represent SEM. Statistical significance was calculated using the One-way ANOVA for each gene with Dunnett’s *post hoc* test for comparisons to the untreated controls (*denotes *P* < 0.05, *n* > 6).

Our working hypothesis is that the Nejire/CBP protein is directly involved in the induction of the alcohol tolerance genes identified in Ghezzi et al. (2013) and that the reason that *nejire* mutants acquire so little alcohol tolerance is because the loss of Nejire/CBP activity blunts the capacity of alcohol to induce these genes. To test this hypothesis, we measured changes in expression of all six genes previously associated with alcohol tolerance and CBP binding (Figure 3, above). As shown in Figure 9, in *nej*^3^ heterozygotes none of the six genes tested are induced by sedation with either benzyl alcohol or ethanol vapor.

In wild-type animals, all three ion channel genes—*slo*, *eag* and *para*— show a significant increase in expression after sedation with either ethanol or benzyl alcohol vapor. The other three genes also showed an overall increase in expression in response to both drugs, but reached significance only for one of the drugs. We believe that these inconsistencies are due variations in the pharmacological dynamics of these two drugs. Nonetheless, in the *nej*^3^ heterozygous mutant, all changes in expression, in all six genes were completely suppressed. These data indicate that alcohol-induced transcriptional activation of six different alcohol-responsive genes rely on a fully functioning CBP protein system.

## Discussion

Alcohol is a central nervous system depressant that slows neural activity and induces sedation. In response the nervous system elicits homeostatic adaptations to counteract the effects. These adaptations often manifest in the form of tolerance and withdrawal symptoms. Tolerance and withdrawal are two key components of alcohol dependence state (Littleton, 1998; Koob and Le Moal, 2001; Ghezzi and Atkinson, 2011). It is becoming increasingly evident that these alcohol-induced neuroadaptations rely on lasting transcriptional changes and are believed to involve coordinate regulation of multi-gene networks. In both flies and mammals, examples of multigenic transcriptional neuroadaptation have been shown, and include amongst others, the restructuring of chromatin states (Ghezzi et al., 2013; Kyzar and Pandey, 2015), the regulation of miRNA expression (Ghezzi et al., 2016; Teppen et al., 2016) and the activation of neuroimmune signaling cascades (Blednov et al., 2012; Troutwine et al., 2016).

Here, we demonstrate that *nejire*, the only *Drosophila* ortholog of the mammalian histone acetyltransferase CBP, mediates the induction of genes to produce functional behavioral tolerance to alcohol. Mutations in *nejire*: (1) block alcohol-induced histone acetylation at an alcohol tolerance gene; (2) block alcohol induction of alcohol tolerance genes; and (3) block alcohol tolerance itself, whereas transgenic induction of *nejire* phenocopies tolerance in alcohol-naive animals. We thus propose that the histone acetyltransferase CBP, is a central regulator of a network of alcohol-responsive genes.

The acronym CBP stands for CREB-binding protein in reference to its first discovered pairing partner, CREB (in recent literature it is also sometimes referred to as CREBBP or KAT3A). However, it is now known that CBP interacts with many hundreds of proteins and is involved in many signaling pathways including cAMP, Notch, hormone, immune, stress response, p53 and cell growth signaling pathways. While CBP is best known as a histone acetyltransferase that regulates gene expression through its effect on chromatin structure, it is more accurate to think of it as a protein acetyltransferase that regulates the activity of a wide variety of proteins. The expression of CBP appears to be tightly regulated and changes in gene activity due to mutations have profound effects on animals. The details of the CBP interactome and the function of CBP in animals are well described in the excellent reviews by Janknecht (2002) and Dancy and Cole (2015).

Monitoring the expression of six alcohol tolerance genes (Figure 9), we observed that a mutation in *nejire* suppressed both ethanol and benzyl alcohol induction of all six genes. For this reason we postulate that CBP is a linchpin coordinate regulator of a network of genes that produce alcohol tolerance. The simplest mechanism that can account for our results is that Nejire/CBP is recruited to these genes by a transcription factor and that it is directly responsible for alcohol induction of gene expression mediated by the acetylation of local histones—albeit additional work will be required to confirm this direct role hypothesis for all six genes. Furthermore, while in Ghezzi et al. (2013) we report that *nejire* gene expression is induced by alcohol sedation, and here we show that *nejire* induction can phenocopy tolerance, it is possible that the relevant mode of alcohol-mediated *nejire*/CBP regulation is post transcriptional. CBP acetyltransferase activity has been proposed to be post-transcriptionally regulated by phosphorylation, acetylation and by metabolism—interestingly, the abundance of acetate itself is altered by ethanol exposure which could affect CBP activity (Janknecht, 2002; Soliman and Rosenberger, 2011).

Despite the complexity of CBP’s interactome, we postulate that the role of CBP in regulating alcohol response genes involves its recruitment to the transcriptional control regions of these genes by the CREB transcription factor. This based on the observation that both *Creb2b* mutants and a *nejire* mutant flatten the alcohol-induced histone acetylation profiles of the *slo* alcohol tolerance gene and simultaneously block the acquisition of alcohol tolerance (see Figure 6A of Wang et al., 2007) and Figure 8 this manuscript). Moreover, CBP is also known to interact with the HDAC Sir2 (Smolik, 2009). Interestingly, down regulation of Sir2 by alcohol has recently been associated with presynaptic changes linked to the development of alcohol tolerance and preference (Engel et al., 2016). The coordinate induction of CBP and suppression of Sir2 by alcohol can dramatically reshape acetylation states.

Modulation of the acetylation states of chromatin regions is a critical component of transcriptional regulation, and as such, it can have a strong impact on the expression profile of a cell or tissue. It is now clear, that chromatin remodeling is also a central component in promoting neuroadaptation to alcohol (Krishnan et al., 2014). Through a tightly controlled balance between acetylation and deacetylation of chromatin regions, the nervous system can fine tune excitability. This dogma seems to hold true in both flies and mammals, as the interplay between HDAC and HAT activity has been shown to control several aspects of the alcohol response, from tolerance, preference and reward in flies (as shown here and in Engel et al., 2016), to the anxiolytic effects of alcohol in mammals (Pandey et al., 2008). In both cases, the histone acetyltransferase CBP is a key player.

## Author Contributions

AG, XL and NSA conceived and designed the experiments; AG, XL, LKL and TPW performed the experiments and analyzed the data. All authors contributed to drafting and revising the work; and approved the final version to be published.

## Funding

This work was funded by National Institute on Alcohol Abuse and Alcoholism (NIAAA) grant to NSA (Grant number #R01AA018037).

## Conflict of Interest Statement

The authors declare that the research was conducted in the absence of any commercial or financial relationships that could be construed as a potential conflict of interest.
