# Supplementary material for: Alcohol-Induced Neuroadaptation Is Orchestrated by the Histone Acetyltransferase CBP
Source: Front Mol Neurosci. 2017 Apr 11;10:103. doi: 10.3389/fnmol.2017.00103 (PMC5387060; doi:10.3389/fnmol.2017.00103)
Supplement: Supplementary file 1 [file DataSheet_1.docx]

Supplementary Material

Alcohol-induced neuroadaptation is orchestrated by the histone acetyltransferase CBP

Alfredo Ghezzi ^1^*, Xiaolei Li ^2^, Linda K. Lew ^2^, Thilini Wijesekera ^2^, Nigel S. Atkinson ^2^*

*** Correspondence:**

Alfredo Ghezzi: alfredo.ghezzi@upr.edu

Nigel S. Atkinson: nsatkinson@austin.utexas.edu

Supplementary Figures:


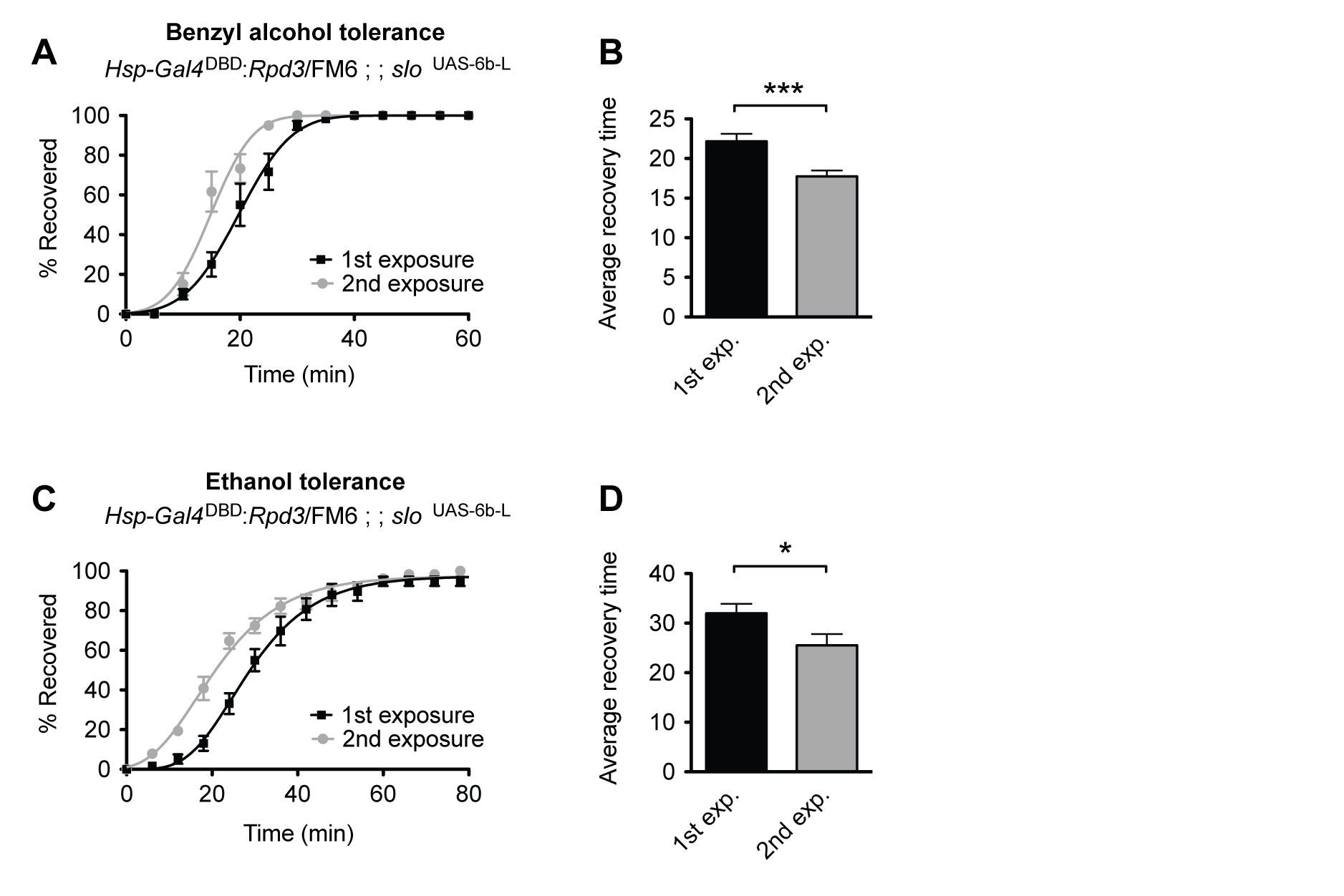


**Supplementary Figure 1.** Insertion of UAS in the *slo* transcriptional control region does not block tolerance to alcohols. A–B) Recovery curves (A) and average-recovery time (B) of uninduced *Hsp-Gal4*^DBD^:*Rpd3*/FM6 ; ; *slo*^UAS-6b-L^ flies recovering from their first and second sedative exposure to benzyl alcohol. Error bars represent SEM (Student's t-test: *** denotes P = 0.0003, n = 60 each). C–D) Recovery curves (C) and average-recovery time (D) of uninduced *Hsp-Gal4*^DBD^:*Rpd3*/FM6 ; ; *slo*^UAS-6b-L^ flies recovering from their first and second sedative exposure to ethanol. Error bars represent SEM (Student's t-test: * denotes P = 0.0324, n = 53 [1st exp]_._, 51 [2nd exp]).

**
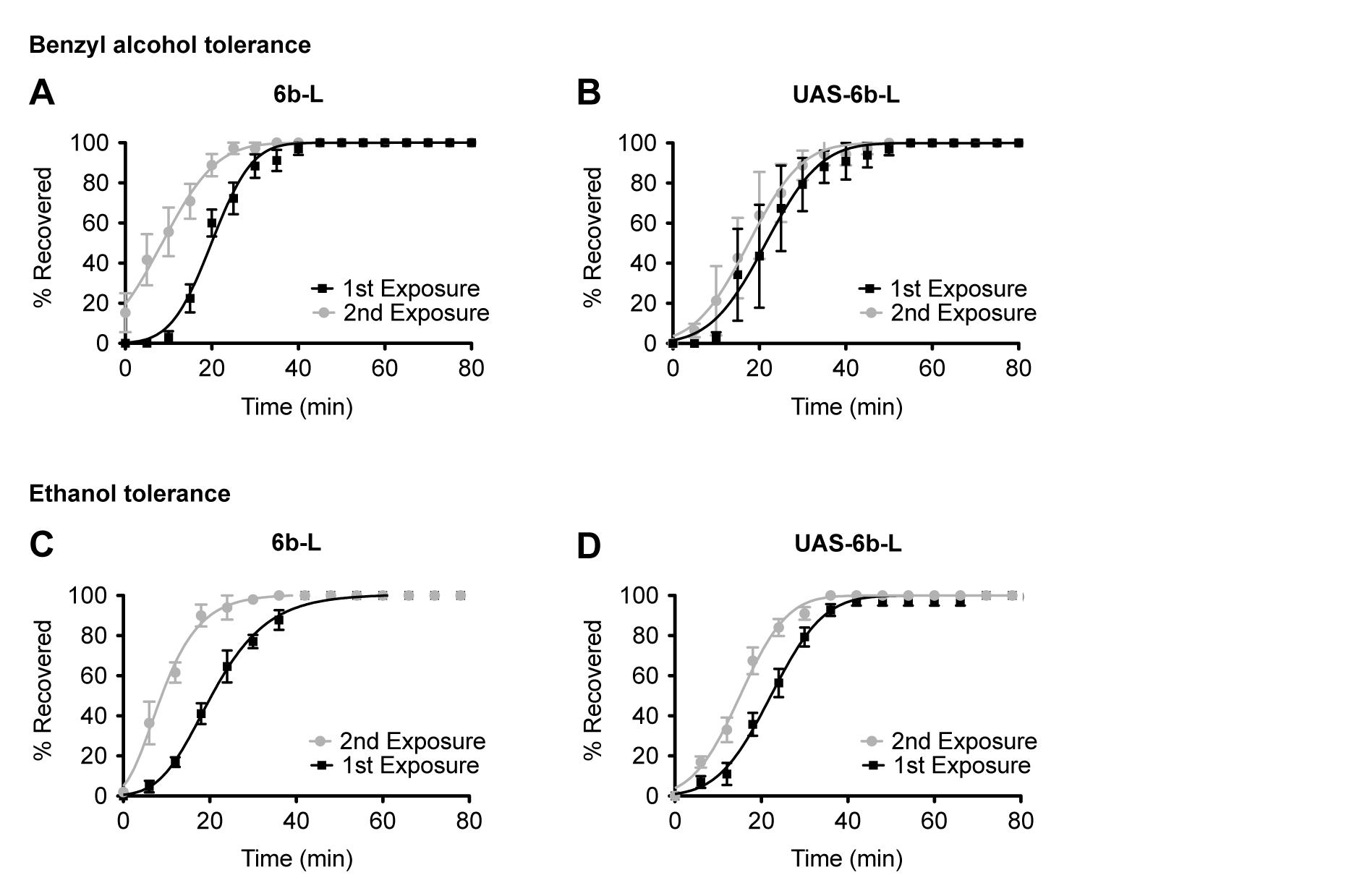
**

**Supplementary Figure 2.** Recovery curves for data presented in Figure 2. Shown are the recovery curves for the experimental groups displayed in Figure 2: the *Hsp-Gal4*^DBD^:*Rpd3*/FM6 ; ; *slo*^6b-L^ animals (6b-L) and in the *Hsp-Gal4*^DBD^:*Rpd3*/FM6 ; ; *slo*^UAS-6b-L^ (UAS-6b-L). All animals were heat treated to activate *Gal4*^DBD^:*Rpd3* expression. The time of heat activation was 30 minutes after the first alcohol treatment. Animals were sedated with ethanol **(A, B)** or benzyl alcohol **(C, D)** and the recovery from one or two sedations was monitored. In each, the black curve represents recovery from a first sedation (1st exposure), whereas the gray curve represents recovery from a second sedation (2nd exposure). The time between sedations was 24 hrs. Error bars represent SEM. The difference in recovery between exposures is presented in the tolerance index (shown in Figure 2).

**
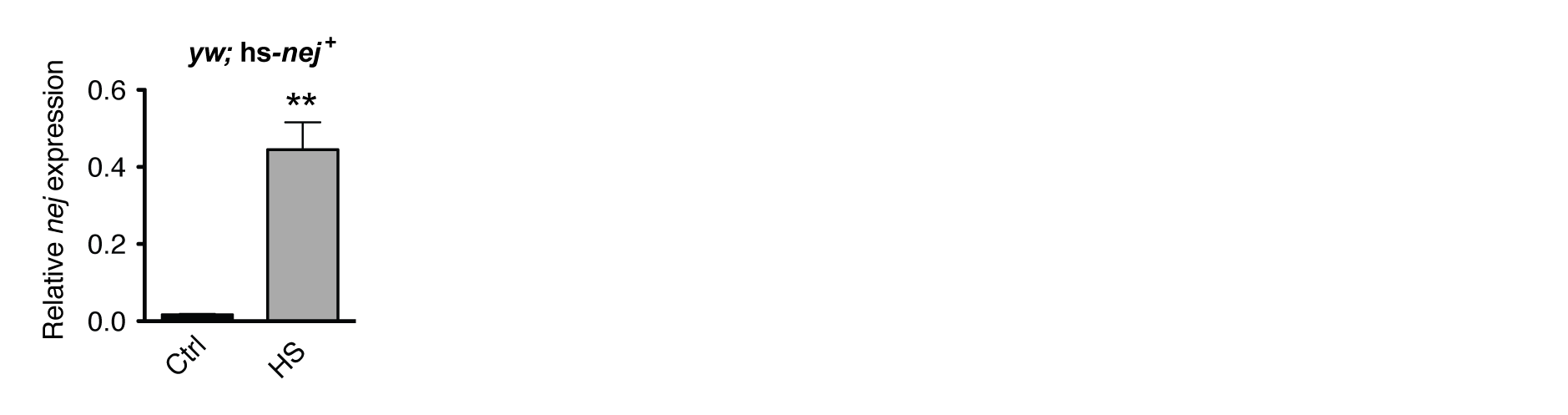
**

**Supplementary Figure 3.** Induction of *nejire* expression using heat-inducible transgene. Shown is the expression of *nejire* mRNA, relative to the expression of the internal control gene (*Cyp1*), 6 hours after a one-hour heat-shock induction of the hs-*nej*^+^ transgene (HS) or the uninduced control (Control). Error bars represent SEM (Student's t-test: ** denotes P < 0.0038, n = 3 [Control], 3 [HS]).
